# More on the Fascinating Characterizations of Mulatu’s Numbers

**DOI:** 10.12688/f1000research.157738.1

**Published:** 2024-10-31

**Authors:** Derebew Nigussie Derso, Ageze Abye Admasu

**Affiliations:** 1 Department of Mathematics, College of Natural and Computational Sciences, Woldia University, Woldia, Ethiopia

**Keywords:** Mulatu’s number; Mulatu’s sequence; γ-Mulatu summable; Mulatu’s series; Mulatu’s characteristics number; generating function.

## Abstract

**Background:**

The discoveries of Mulatu’s numbers, better known as Mulatu’s sequence, represent revolutionary contributions to the mathematical world. His best-known work is Mulatu’s sequence, in which each new number is the sum of the two preceding numbers. When various operations and manipulations are performed on the numbers in this sequence, beautiful and incredible patterns begin to emerge. This study aimed to identify novel characterizations of Mulatu’s numbers.

**Methods:**

This study employed a multi-faceted approach to investigate characterizations of Mulatu’s numbers. Mathematical proof techniques such as principle of mathematical induction, proof by contradiction and direct proof were utilized to substantiate findings. GNU Octave (version 9, (1)) software was applied to verify the existence, classify, and conduct computational investigations of findings prior to formal proofs.

**Results:**

In this study, we provided several characterizations of Mulatu’s numbers. We also investigated the properties and patterns of these fascinating numbers. Moreover, we have also shown that, similar to Fibonacci’s numbers, Mulatu’s numbers also give the so-called golden ratio, which is most applicable in numerical optimization. Furthermore, we formulated a relation among Mulatu’s numbers, Fibonacci numbers, and Lucas numbers. Finally, we provided a generating function for the Mulatu numbers.

**Conclusions:**

In this study, we uncovered novel characterizations of Mulatu’s numbers and introduced a generating function for them. We investigated relationship between Mulatu’s numbers and the golden ratio. The results discussed offer valuable insights and enhance our understanding of their properties. Furthermore, these findings play a vital role in the boarder context of mathematical sequences, contributing significantly to the field.

## Introduction

Mulatu numbers are a recently introduced sequence by Mulatu Lemma, a Professor of Mathematics at Savannah State University in Savannah, Georgia. ^2, 3^ These numbers have been introduced in different studies. ^2– 5^ Mulatu’s numbers defined as 4, 1, 5, 6, 11, 17, 28, 45, 73, … . Mathematically, such sequence can be written as follow.

$$M_{n}=\left\{ \begin{array}{ll} 4 & \text{if}\;n=0\\ 1 & \text{if}\;n=1\\ M_{n-1}+M_{n-2} & \text{if}\;n>1 \end{array}\right.$$

Mulatu Lemma’s work has sparked curiosity in many scholars, encouraging them to dive deeper into this fascinating field. He’s especially recognized for a special sequence of numbers that bears his name, known for its intriguing patterns. Mulatu’s contributions continue to inspire and engage anyone interested in the beauty of mathematics. ^6, 7^

Mulatu’s sequence is a fascinating series of numbers where each new term is found by adding the two before it. It’s the latest example of a recursive sequence, meaning each term is built from the previous ones. Taking a closer look at Mulatu’s sequence opens the door to a wealth of fascinating patterns and mathematical properties. ^6^ Over the years, researchers have discovered many interesting trends within it. For instance, the Fibonacci sequence starts with 0, 1, 1, 2, 3, 5, 8, 13, 21, 34, 55, and continues on, while the Lucas sequence begins with 2, 1, 3, 4, 7, 11, 18, 29, 47, 76, 123, and grows from there. ^8^ Both of these sequences showcase the elegance and intricacy of numbers.

Previous works also discussed many of the Fibonacci and Luca numbers, ^2, 3, 8– 10^ but only some characterizations of Mulatu’s numbers. ^2, 3, 6, 7^ Thus, in this paper, we focused on these less studied numbers, Mulatu’s numbers. We have produced some fascinating results on Mulatu’s numbers and on the interrelationships between Fibonacci’s and Lucas’s numbers. We are interested in the generic area of gradient-free optimization when derivative information for the function is unavailable or calculation of the derivatives is computationally difficult, and the Golden section search method is one such algorithm that uses the Golden ratio as an input. ^11^ Hence, in addition, we related the Mulatu sequence with the so-called golden ratio, which is most applicable in numerical optimization, specifically to find an approximate optimizer with a small error.

## Methods

In this study, we applied various mathematical proof techniques, including the principle of mathematical induction, proof by contradiction, and direct proof, to investigate multiple characterizations. Additionally, we utilized GNU Octave (version 9, ^1^) software to formulate conjectures, verify existence, classify results, and conduct numerical analyses prior to presenting formal proofs. This combination of theoretical and computational approaches enabled a comprehensive exploration of Mulatu numbers.

### Characterizations of Mulatu’s Numbers

Lemma 1. Any two consecutive Mulatu numbers are relatively prime.

Theorem 1. Adding any ten consecutive Mulatu numbers together will always result in a number that is divisible by 11.

Proof. Let $q=\sum_{n=i}^{i+9}M_{n}$ for any number i . We then show that q is divisible by 11 .

Using the definition of Mulatu numbers, we have $M_{i+j}=xM_{i}+yM_{j}\;$ for j≥2 in the set of natural numbers, where x and y are given below.

**Table:** 

j	x	y
2	1	1
3	1	2
4	2	3
5	3	5
6	5	8
7	8	13
9	21	34


$$q=\sum_{n=i}^{i+9}M_{n}=M_{i}+M_{i+1}+M_{i+2}+M_{i+3}+\ldots +M_{i+9}=55M_{i}+88M_{i+1}.$$

This completes the proof.

Theorem 2. Multiplying any Mulatu’s number by two and subtracting the next Mulatu’s number in the sequence for a number greater than or equal to two will result in the answer being Mulatu’s number, that is, $2M_{n}-M_{n+1}=M_{n-2}$ , n≥2.

Proof. We use induction on n . The formula holds for n=2 as we have,

$M_{2}=5$ , $M_{n}=6$ , $2\times 5-6=4=M_{0}$ . Assume that the formula holds true for n=k that is, $2M_{k}-M_{k+1}=M_{k-2}$ , k≥2 . This assumption leads to $\;2M_{n}-M_{n+1}=M_{n-2}$ , n≥2.

Theorem 3. When adding consecutive, even-positioned Mulatu numbers beginning with $\;M_{2i}$ , for i≥1 , the result is a number that is one less than the Mulatu number succeeding the last Mulatu number in the sum. This provides a general formula for a simple way to find the sum of any finite even-positioned Mulatu number.

$$\sum_{i=1}^{n}\;M_{2i}=M_{2n+1}-1.$$

Proof. We use induction on n . It is true for n=1 as $M_{2}=5=M_{3}-1.$

Assume it holds for 2≤k≤n . That is $\sum_{i=1}^{k}\;M_{2i}=M_{2k+1}-1.$ Now,

$$\begin{array}{c} \sum_{i=1}^{k}M_{2i}=\sum_{i=1}^{k}M_{2i}+M_{2k+2}+M_{2k+4}+M_{2k+6}+\ldots +M_{2n-2}+M_{2n}\\ \;=M_{2k+1}-1+M_{2k+2}+M_{2k+4}+M_{2k+6}+\ldots +M_{2n-2}+M_{2n} \end{array}$$

However,

$$\;M_{2k+1}=M_{2k+3}-M_{2k+2},M_{2k+3}=M_{2k+5}-M_{2k+4},\ldots ,M_{2k+2j+1}=M_{2k+2j+3}-M_{2k+2j+2},$$

for any natural number j . This implies

$$M_{2k+2\left(n-k\right)-1}=M_{2n-1}=M_{2k+2\left(n-k\right)+1}-M_{2k+2\left(n-k\right)}=M_{2n+2}-M_{2n}.$$

So, we are done.

Theorem 4. When adding consecutive, odd-positioned Mulatu’s numbers beginning with $\;M_{1}$ , only this time, the result is a number that is four less than the Mulatu number following the last even number in the sum. This provides a general formula for a simple way to find the sum of any finite odd-positioned Mulatu number.

$$\sum_{i=1}^{n}M_{2i-1}=M_{2n}-4.$$

Proof. We use induction on n .

Theorem 5. When any four consecutive numbers in the Mulatu sequence are considered, the difference between the squares of the two numbers in the middle is equal to the product of the two outer numbers. Mathematically,

$${M_{n+1}}^{2}-{M_{n}}^{2}=M_{n-1}M_{n+2},n\geq 1.$$

Corollary 1. For n≥1 , $2M_{n}+M_{n-1}=M_{n+2}.$

Proof. By using Theorem 5 above and the relation $\;M_{n+1}=M_{n}+M_{n-1}$ , the result follows.

Theorem 6. Adding any number of consecutive Mulatu numbers will result in a number that is one less than the Mulatu number of two places beyond the last summand. This provides a general formula for a simple way to find the sum of any number of Mulatu numbers.

$$\sum_{i=0}^{n}M_{i}=M_{n+2}-1.$$

Theorem 7. Let $\;M_{n}$ , $F_{n}$ and $L_{n}$ be the Mulatu’s, Fibonacci’s, and Lucas’s numbers for each n≥0 . The number $\left(M_{n}+M_{n+3}\right)\left(F_{n}+F_{n+3}\right)\left(L_{n}+L_{n+3}\right)$ is divisible by 8.

Proof. We use induction on n .

For $n=0,M_{0}+M_{3}=10,F_{0}+F_{3}=2$ and $L_{0}+L_{3}=6$ . This implies that

$\left(M_{0}+M_{3}\right)\left(F_{0}+F_{3}\right)\left(L_{0}+L_{3}\right)=120\;$ is divisible by 8.

Assume that the statement holds for n=k . As $\;M_{k}+M_{k+3}=2\left(M_{k}+M_{k+1}\right)$ , we have

$$M_{k+1}+M_{k+4}=2\left(M_{k}+2M_{k+1}\right).$$

Thus $,2\;|\left(M_{k+1}+M_{k+4}\right)$ . Similarly, $2\;|\left(F_{k+1}+F_{k+4}\right)$ and $\;2|\left(L_{k+1}+L_{k+4}\right)$ .

Hence, the theorem holds by the principle of mathematical induction.

Theorem 8. Half of the sum of any two even consecutive Mulatu numbers yields Mulatu’s number preceding the larger one. i.e.,

$$\frac{1}{2}\left(M_{3n}+M_{3\left(n+1\right)}\right)=M_{3\left(n+1\right)-1}.$$

Proof. We use induction on n .

For $n=0,\frac{1}{2}\left(M_{0}+M_{3}\right)=5=M_{2}.$ Let it be true for n=k . That is

$$\frac{1}{2}\left(M_{3k}+M_{3\left(k+1\right)}\right)=M_{3\left(k+1\right)-1}.$$

This assumption with the definition of Mulatu’s sequence leads:

$\frac{1}{2}\left(M_{3n}+M_{3\left(n+1\right)}\right)=M_{3\left(n+1\right)-1},$ for all n.

Definition 1. a) Two natural numbers are said to be Mulatu summable if their sum is a Mulatu number.

b) A natural number k is said to be γ -Mulatu summable if a natural number γ≥2 exists such that γk is a Mulatu number.

Example: 2 and 3 are Mulatu summable, but 3 and 5 are not Mulatu summable. Moreover, 1 is γ -Mulatu summable for all $\;\gamma =M_{n}\neq 1$ , where $M_{n}$ is the Mulatu number and 2 is γ -Mulatu summable for γ=2,3,14,.…

Definition 2. Let γ be the smallest natural number such that a natural number k is γ -Mulatu summable. Then, γ is said to be the Mulatu characteristic of k , denoted by $m^{*}\left(k\right).$

Example: $\;m^{*}\left(1\right)=4$ , $m^{*}\left(2\right)=2$ , $m^{*}\left(4\right)=7$ and so on.

Lemma 2. The Mulatu characteristics neither preserve nor reverse any inequality.

Proof. Let k and n be natural numbers with k<n . Suppose that the Mulatu characteristic either preserve or reverse an inequality. That is, the Mulatu characteristics preserve or reverse an inequality. Thus, it must preserve or reverse the inequality k<n . Thus, $m^{*}\left(k\right)<m^{*}\left(n\right)$ or $m^{*}\left(k\right)>m^{*}\left(n\right)$ for each k<n .

Neither is true because, for instance, 2<3 but $\;m^{*}$ (2) = 2 = $m^{*}$ (3). For the remaining inequalities, we can see counter examples 1<2 but $\;m^{*}\left(1\right)=4>1=m^{*}\left(2\right)$ ; and 3<4 , with

$\;m^{*}\left(3\right)=2<7=m^{*}\left(4\right)\;$ . Thus, the negation of the given statement is not true, and this completes the proof.

Theorem 9. Let k and n be natural numbers such that k<n , then

$$km^{*}\left(k\right)\leq {\mathrm{nm}}^{*}\left(n\right).$$

Proof. Suppose not. That is $km^{*}\left(k\right)\leq {\mathrm{nm}}^{*}\left(n\right)$ for all k<n.

Dividing both sides by $km^{*}\left(n\right)$ gives

$\frac{\;m^{*}\left(k\right)}{\;m^{*}\left(n\right)}>\frac{n}{k}$ , ∀k<n.

This implies

$m^{*}\left(k\right)>m^{*}\left(n\right)$ > m∗(n), ∀k<n , contradicting Lemma 2.

Theorem 10. Let $\gamma_{i}\geq 2$ and $\gamma_{i+1}>2\;$ for i≥0 be consecutive natural numbers such that 2 is $\gamma_{i}$ -Mulatu summable and is $\gamma_{i+1}$ -Mulatu summable, respectively, and let $\gamma_{i}$ and $\gamma_{i+1}$ be Mulatu summable.

Proof. We use induction on i .

For $i=0,\gamma_{0}=2,\gamma_{1}=3\;$ and $\;\gamma_{0}+\gamma_{1}=5=M_{2}$ . Assuming it holds for i=k and Theorem 8, we get

$$\gamma_{k+1}+\gamma_{k+2}=\frac{1}{2}\left(2\gamma_{k+1}+2\gamma_{k+2}\right),$$

which is a Mulatu’s number.

Example: What is the successor to γ=3 and γ=54 in Theorem 10?

The answer is 14 and 225 respectively.

### Relationship with the Golden Ratio

The golden ratio is defined by taking a line segment and dividing it into two parts: the longer part (L) and the shorter part (S). The ratio of L to S is the same as the ratio of the entire line segment to L. Letting this ratio x , leads $\;x=1+\frac{1}{x}$ , whose solution is the golden ratio, ϕ=1.6180339887… .

The golden ratio pops up in so many places, from mathematics to art and nature. It’s like a secret thread that ties together beauty and balance. People often find it aesthetically pleasing, whether it’s in a stunning painting, an elegant building, or the way a sunflower blooms. ^8, 10^

Furthermore, what’s particularly cool is that when we simplify the reciprocal of ϕ , it turns out to be just one less than ϕ itself. This means $\;\phi -\frac{1}{\phi }=1$ . It’s quite special that ϕ and its reciprocal are two numbers for which both their difference and their product equal one.

Surprisingly, Mulatu numbers have a fascinating connection to the golden ratio. When we divide one Mulatu number by the one before it, the result gets closer and closer to ϕ as the numbers increase. This means that Mulatu numbers can be used to calculate the golden ratio.

Some of such ratios are:

$$\begin{array}{c} \frac{M_{6}}{M_{5}}=1.6470588235\\ \frac{M_{7}}{M_{6}}=1.6071428571\\ \frac{M_{8}}{M_{7}}=1.6222222222..\\ \ldots \\ \frac{M_{25}}{M_{24}}=1.6180339884\\ \frac{M_{26}}{M_{25}}=1.6180339889\\ \frac{M_{27}}{M_{26}}=1.6180339887\\ \ldots \end{array}$$

This implies that $\;\lim_{n\to \infty }\frac{M_{n+1}}{M_{n}}=\phi$ .

Conversely, if Mulatu’s number is divided by the succeeding Mulatu number, the result is close to the reciprocal of ϕ . Again, the larger the two numbers used, the closer the result to the reciprocal of ϕ .

### Generating Functions for the Mulatu’s Number

In this section, we give a generating function for the sequence $\;{\left\{M_{n}\right\}}_{n=0}^{\infty }$ .

Definition 3. The series $\sum_{n=0}^{\infty }M_{n}\;$ is called Mulatu’s series.

Theorem 11. Mulatu’s series is divergent.

Proof. Using the ratio test, $\lim_{n\to \infty }\frac{M_{n+1}}{M_{n}}=1.618>1.$ Thus, it diverges.

Theorem 12.
$f\left(x\right)=\sum_{n=0}^{\infty }M_{n}x^{n}=\frac{4-3x}{1-x-x^{2}}$ is a generating function for the Mulatu’s sequence ${\left\{M_{n}\right\}}_{n=0}^{\infty }.$

Proof. Let $\;f\left(x\right)=\sum_{n=0}^{\infty }M_{n}x^{n}$ .

Claim: $f\left(x\right)=\frac{4-3x}{1-x-x^{2}}$

Using the properties of Mulatu’s numbers, specifically, $\;M_{n+2}=M_{n}+M_{n+1},$ we have

$$\begin{array}{c} f\left(x\right)=\sum_{n=0}^{\infty }M_{n+2}x^{n}=\sum_{n=0}^{\infty }M_{n+1}x^{n}+\sum_{n=0}^{\infty }M_{n}x^{n}\\ \;=\sum_{n=1}^{\infty }M_{n}x^{n-1}+f\left(x\right) \end{array}$$

This implies,

$$\sum_{n=2}^{\infty }M_{n}x^{n}=x\sum_{n=1}^{\infty }M_{n}x^{n}+x^{2}f\left(x\right)$$

That is

$$-M_{0}-M_{1}x+f\left(x\right)=-M_{0}x+\mathrm{xf}\left(x\right)+x^{2}f\left(x\right).$$

Substituting $M_{0}$ and $\;M_{1}$ completes the proof.

## Conclusion

In this work, we found many fascinating new characterizations of Mulatu numbers. We produced relationships among Mulatu’s, Fibonacci’s, and Lucas’s numbers and with the set of natural numbers as well. Namely, we categorized two natural numbers or a natural number as the Mulatu summable or γ -Mulatu summable, respectively. Moreover, we have also shown that, similar to Fibonacci’s numbers, Mulatu’s numbers give the so-called golden ratio, which is most applicable in numerical optimization. Finally, we provide a generating function for the Mulatu numbers. These findings play a crucial role in the realm of mathematical sequence world.

### Ethical declaration

We hereby declare that the information provided above is accurate, and that this research was conducted in compliance with all applicable ethical guidelines and institutional policies. Since this study did not involve any human or animal participants, there was no need for ethical approval or consent.

### Declaration of originality

We declare that the research presented in this paper is our original work. This work has not been submitted for any other degree or qualification, and all the sources and references used have been appropriately acknowledged.

## Data Availability

No data are associated with this article. Since it is a study on a mathematical theory, there is no extended data used.
