# Supplementary material for: More on the Fascinating Characterizations of Mulatu’s Numbers
Source: F1000Res. 2026 Feb 24;13:1306. Originally published 2024 Oct 31. [Version 3] doi: 10.12688/f1000research.157738.3 (PMC12963824; doi:10.12688/f1000research.157738.3)
Supplement: Peer review history

# Reviewer report: Reviewer response for version 3

**DOI:** 10.5256/f1000research.189018.r461838

**Published:** 2026-03-05

**Authors:** Hayder R. Hashim

**Affiliations:** 1 University of Kufa, Kufa, Najaf Governorate, Iraq

The current version of the manuscript is much better than the previous version, however it still has some issues that need to be considered. 

 1. The abstract should not be written in this current form with different parts of Background, Methods, conclusion,.... It should be in one paragraph summarizing the main problem of the paper with the used tools. 

 2. The fourth paragraph in the introduction is cited as, .... further illustrates the ongoing effort to uncover deeper properties, for more see Refs. 8–14. But this citation is different from the other used forms. 

 3. Theorem 4 should be proved, as well as Corollary 1.

 4. The Example below of Theorem 10 should be numbered with bold letters. 

 5. In general, In its current form, the manuscript reads more like extended lecture notes than a polished research article. Several arguments are presented in a step-by-step instructional manner, with lengthy proofs and limited high-level guidance. For a journal paper, the results should be more concise and focused on the main ideas, with clearer separation between definitions, key lemmas, and central results.

Is the work clearly and accurately presented and does it cite the current literature?

Partly

If applicable, is the statistical analysis and its interpretation appropriate?

Not applicable

Are all the source data underlying the results available to ensure full reproducibility?

No source data required

Is the study design appropriate and is the work technically sound?

Yes

Are the conclusions drawn adequately supported by the results?

Yes

Are sufficient details of methods and analysis provided to allow replication by others?

Yes

Reviewer Expertise:

Number theory

I confirm that I have read this submission and believe that I have an appropriate level of expertise to confirm that it is of an acceptable scientific standard, however I have significant reservations, as outlined above.

# Reviewer report: Reviewer response for version 3

**DOI:** 10.5256/f1000research.189018.r461842

**Published:** 2026-03-05

**Authors:** FUNDA TAŞDEMİR

**Affiliations:** 1 Yozgat Bozok University, Yozgat, Turkey

The authors have revised the manuscript in response to the reviewers’ comments and addressed the main points raised. The manuscript has been clarified in several places, examples have been added, and minor technical issues have been corrected. Although the overall improvement is moderate, the paper has benefited from the revision process. In its current form, I consider the manuscript acceptable for indexing.

Is the work clearly and accurately presented and does it cite the current literature?

Partly

If applicable, is the statistical analysis and its interpretation appropriate?

Not applicable

Are all the source data underlying the results available to ensure full reproducibility?

Partly

Is the study design appropriate and is the work technically sound?

Yes

Are the conclusions drawn adequately supported by the results?

Yes

Are sufficient details of methods and analysis provided to allow replication by others?

Yes

Reviewer Expertise:

Number theory, special sequences

I confirm that I have read this submission and believe that I have an appropriate level of expertise to confirm that it is of an acceptable scientific standard.

# Reviewer report: Reviewer response for version 2

**DOI:** 10.5256/f1000research.178948.r407779

**Published:** 2025-09-18

**Authors:** FUNDA TAŞDEMİR

**Affiliations:** 1 Yozgat Bozok University, Yozgat, Turkey

In this paper, the authors investigate Mulatu’s sequence, a type of linear recurrence sequence, with an emphasis on its basic algebraic properties.

 The paper is easy to understand manner. While the identities presented are a useful starting point, they are not sufficient to fully characterize the sequence. I recommend that the authors include additional properties or identities to strengthen the mathematical foundation of the paper.

 The paper should be well developed. It is not appropriate to index it in its current form. In my opinion, the paper could be accepted for indexing after a major revision according to the following suggestions:

  

- The introduction needs to be expanded by situating the study within the context of relevant literature and emphasizing its significance and contributions.
- The manuscript would benefit from replacing informal terms like “fascinating” with more precise and objective language to maintain an academic tone.
- A more thorough and detailed exposition of the connection between Mulatu numbers, Fibonacci numbers, and the golden ratio is necessary to strengthen the mathematical foundations of the study.
- In general, the theorems and proofs should be presented in a more formal and mathematically rigorous manner.
- The manuscript refers to GNU Octave for numerical verification, yet lacks corresponding results. Presenting numerical evidence would reinforce the validity of the findings.
- Page 4, proof of Lemma 1, should be “For *n=0,* .”
- The identities/properties used should be either explicitly stated prior to their use or cited from relevant literature. (For example: Page 4, proof of the Theorem 1)
- References should be improved and organized. (For example: Reference 1 (Lemma M,…) and Reference 6 (Mulatu L:)).
- To improve the logical flow, intermediate computational steps should be explicitly presented. (For instance: Page 6, proof of the Theorem 8)

Is the work clearly and accurately presented and does it cite the current literature?

Partly

If applicable, is the statistical analysis and its interpretation appropriate?

Not applicable

Are all the source data underlying the results available to ensure full reproducibility?

Partly

Is the study design appropriate and is the work technically sound?

Yes

Are the conclusions drawn adequately supported by the results?

Yes

Are sufficient details of methods and analysis provided to allow replication by others?

Yes

Reviewer Expertise:

Number theory, special sequences

I confirm that I have read this submission and believe that I have an appropriate level of expertise to confirm that it is of an acceptable scientific standard, however I have significant reservations, as outlined above.

# Response

**Published:** 2025-09-22

**Authors:** AGEZE ADMASU

**Affiliations:** Mathematics, Woldia University, Woldia, Amhara, Ethiopia

DEAR PROFESSOR FUNDA TAŞDEMİR

 We are in the process of addressing the comments given and will submit the revised manuscript as soon as possible.

 However, we would like to clarify that the GNU Octave were conducted to assist in the conceptualization and formulation of the conjectures presented in the paper, not for analysis or illustration of results.

 Sincerely,

 Ageze Abye Admasu

 Woldia University, Ethiopia

# Reviewer report: Reviewer response for version 2

**DOI:** 10.5256/f1000research.178948.r405228

**Published:** 2025-09-05

**Authors:** Hakan Akkuş

**Affiliations:** 1 Erzincan Binali Yıldırım University, Erzincan, Turkey

Dear Editor,

 I read the article from beginning to end. I can say that the links in the article are nice and clear. Furthermore, the arrangements are technically correct and have a logical structure with a beginning, middle, and end. I checked the procedures, and everything appears to be correct. I hope it will be of interest to the journal's readers. For the reasons mentioned above, I would recommend that you accept the article for the journal. However, I recommend that authors consider the following revisions before the Editor makes a decision:

 *** I recommend including some previous work in the abstract, such as the generating functions and sum formulas of mulatu numbers.

 *** Mulatu numbers are a special case of Fibonacci numbers, and I believe that mentioning Fibonacci numbers and their applications in the introduction will enhance the main message of the article and motivate readers.

 *** I recommend reconsidering the definitions, theorems, and auxiliary theorems.

 *** I find the references insufficient and recommend the addition of the following works.

 Recommendation: Minor revision

Is the work clearly and accurately presented and does it cite the current literature?

Yes

If applicable, is the statistical analysis and its interpretation appropriate?

I cannot comment. A qualified statistician is required.

Are all the source data underlying the results available to ensure full reproducibility?

Partly

Is the study design appropriate and is the work technically sound?

Yes

Are the conclusions drawn adequately supported by the results?

Yes

Are sufficient details of methods and analysis provided to allow replication by others?

Yes

Reviewer Expertise:

Number theory, Algebra, Applied mathematic

I confirm that I have read this submission and believe that I have an appropriate level of expertise to confirm that it is of an acceptable scientific standard, however I have significant reservations, as outlined above.

## References

[1] : Analytical Characterization of Self-Similarity in k-Cullen Sequences Through Generating Functions and Fibonacci Scaling. *Fractal and Fractional* .2025;9(6) : 10.3390/fractalfract9060380 10.3390/fractalfract9060380

[2] : Properties of Generalized Bronze Fibonacci Sequences and Their Hyperbolic Quaternions. *Axioms* .2024;14(1) : 10.3390/axioms14010014 10.3390/axioms14010014

[3] : Copper ratio obtained by generalizing the Fibonacci sequence. *AIP Advances* .2024;14(7) : 10.1063/5.0207147 10.1063/5.0207147

[4] : Fibonacci and Lucas Numbers with Applications.2001; 10.1002/9781118033067 10.1002/9781118033067

[5] : On the Fibonacci k-numbers. *Chaos, Solitons & Fractals* .2007;32(5) : 10.1016/j.chaos.2006.09.022 1615-1624 10.1016/j.chaos.2006.09.022

[6] : On Thabit and Williams numbers base b as sum or difference of Fibonacci and Mulatu numbers and vice versa. *Afrika Matematika* .2025;36(1) : 10.1007/s13370-025-01266-0 10.1007/s13370-025-01266-0

[7] : Mulatu Numbers as Products of Three Generalized Lucas Numbers. *Mathematica Pannonica* .2024;30_NS4(2) : 10.1556/314.2024.00014 142-161 10.1556/314.2024.00014

# Response

**Published:** 2025-09-06

**Authors:** AGEZE ADMASU

**Affiliations:** Mathematics, Woldia University, Woldia, Amhara, Ethiopia

Dear Professor Hakan Akkus,

 Thank you for your valuable feedback on our manuscript, “More on the Fascinating Characterizations of Mulatu’s Numbers”. We have carefully considered all of your comments and suggestions. We agree with your assessment and will revise our manuscript in accordance with your recommendations.

 We believe that these revisions will significantly improve the quality and clarity of our paper. We will submit the revised version as soon as it is complete.

 Thank you again for your time and expertise.

 Sincerely,

 Ageze Abye Admasu

 Department of Mathematics, Woldia University, Ethiopia

# Reviewer report: Reviewer response for version 2

**DOI:** 10.5256/f1000research.178948.r382531

**Published:** 2025-06-09

**Authors:** Kunle Adegoke

**Affiliations:** 1 Obafemi Awolowo University, Ife, Osun, Nigeria

The authors present results for a particular case of a well-known sequence of numbers, the generalized Fibonacci sequence, also called the Gibonacci sequence. Since all the results can be obtained immediately by substituting the starting values G0=4 and G1=1 in the known general results, the results in this manuscript are trivial. I do not recommend indexing in F1000Research.

Is the work clearly and accurately presented and does it cite the current literature?

Yes

If applicable, is the statistical analysis and its interpretation appropriate?

Not applicable

Are all the source data underlying the results available to ensure full reproducibility?

No source data required

Is the study design appropriate and is the work technically sound?

Yes

Are the conclusions drawn adequately supported by the results?

Yes

Are sufficient details of methods and analysis provided to allow replication by others?

Yes

Reviewer Expertise:

NA

I confirm that I have read this submission and believe that I have an appropriate level of expertise to state that I do not consider it to be of an acceptable scientific standard, for reasons outlined above.

# Reviewer report: Reviewer response for version 2

**DOI:** 10.5256/f1000research.178948.r382539

**Published:** 2025-06-02

**Authors:** Merve Güney Duman

**Affiliations:** 1 Sakarya University of Applied Sciences, Sakarya, Turkey

In this manuscript, some properties of Mulatu’s numbers are given. Many of these properties are either known or have obvious proofs.

 There are too many deficiencies in the presented manuscript.

 Proof methods were chosen incorrectly.

 Very simple identities were given.

 Some proofs were not given.

 There are format errors.

 The presentation style of the manuscript is not understandable.

 Introduction should be expanded.

 References should be improved.

 Some intermediate operations should be given.

 There are too many spelling and format errors.

  The article must be well developed. It is not appropriate to publish it in its current form.

Is the work clearly and accurately presented and does it cite the current literature?

No

If applicable, is the statistical analysis and its interpretation appropriate?

Partly

Are all the source data underlying the results available to ensure full reproducibility?

Partly

Is the study design appropriate and is the work technically sound?

No

Are the conclusions drawn adequately supported by the results?

No

Are sufficient details of methods and analysis provided to allow replication by others?

Partly

Reviewer Expertise:

Number theory, Diophantine equations, Special sequences, etc.

I confirm that I have read this submission and believe that I have an appropriate level of expertise to state that I do not consider it to be of an acceptable scientific standard, for reasons outlined above.

# Reviewer report: Reviewer response for version 2

**DOI:** 10.5256/f1000research.178948.r382535

**Published:** 2025-05-28

**Authors:** Hayder R. Hashim

**Affiliations:** 1 University of Kufa, Kufa, Najaf Governorate, Iraq

This article mainly gives nice results to a linear recurrence sequence called  Mulatu’s sequence. 

 The article is well written, but I suggest to improve it as follows:

 - This sequence is a linear recurrence sequence, so you should recall the concept of this type of such sequences regarding to their definition, kinds, and properties. 

 - In the abstract, you mentioned that the numbers of this sequences represent  revolutionary contributions to the mathematical world. What are these contributions to the mathematical world? Can you recall some of the applications to this sequence? Why is it important? Please improve the introduction with the answers of those questions. 

 - In the proof of Lemma 1:" For n=0, gcd(M0,M1)=gcd(41)=1." should be "For n=0, gcd(M0,M1)=gcd(4,1)=1."

 - In Theorem 1"Adding any ten consecutive Mulatu numbers together will always result in a number that is divisible by 11." Can you provide examples satisfying the theorem, and not satisfying the theorem in case of adding more or less that 10  consecutive Mulatu numbers?

 -Relationship with the Golden Ratio. You supposed that limit of M_{n+1}/M_{n}= 1.618...., can you provide a proof for it.

Is the work clearly and accurately presented and does it cite the current literature?

Partly

If applicable, is the statistical analysis and its interpretation appropriate?

Not applicable

Are all the source data underlying the results available to ensure full reproducibility?

No source data required

Is the study design appropriate and is the work technically sound?

Yes

Are the conclusions drawn adequately supported by the results?

Yes

Are sufficient details of methods and analysis provided to allow replication by others?

Yes

Reviewer Expertise:

Number theory

I confirm that I have read this submission and believe that I have an appropriate level of expertise to confirm that it is of an acceptable scientific standard, however I have significant reservations, as outlined above.

# Response

**Published:** 2025-09-22

**Authors:** AGEZE ADMASU

**Affiliations:** Mathematics, Woldia University, Woldia, Amhara, Ethiopia

Dear Professor Hayder R. Hashim

 I would like to extend my sincere gratitude for your insightful comments and valuable feedback on my article. Your suggestions have been incredibly helpful in improving the quality of our work. I truly appreciate the time and effort you dedicated to reviewing it. We will submit the revised manuscript as soon as possible.

 Sincerly,

 Ageze Abye Admasu

 Woldia University, Ethiopia

# Reviewer report: Reviewer response for version 1

**DOI:** 10.5256/f1000research.173234.r358140

**Published:** 2025-02-17

**Authors:** Priyabrata Mandal

**Affiliations:** 1 Manipal Institute of Technology, Manipal, India

**Title and Abstract:**

- The title is appropriate and reflects the paper's content.
- The abstract is clear but somewhat not standard. The phrase "beautiful and incredible patterns" is informal for an academic paper. A more precise statement would improve clarity.

**Introduction:**

- The introduction does not adequately define the novelty of the work. The discussion on the relationship between Mulatu numbers, Fibonacci numbers, and the golden ratio should be more structured.

**Mathematical Rigor and Errors:**

- Lemma 1 states, "Any two consecutive Mulatu numbers are relatively prime." A proof or reference is missing.
- In the proof of Theorem 1, the table for j,x, and y is completely unnecessary. The author should identify explicitly what those x and y actually mean. By this, I mean to show that M _i+j_=F _j-1 _M _i_ + F _j_ M _i+1_, where F _n_ denotes the nth Fibonacci number. 
- The proof of Theorem 2 contains an induction step that is not fully justified. There is no need for induction here; just use the recurrence relation for M _n_​​​​​​.
- In Theorem 3, the proof is poorly structured, making it difficult to follow the logic behind the claim. No need for induction; note that M _2n_=(M _2n+1 _- M _2n-1_), and proceed.
- Several theorems, such as Theorems 6 and 7, rely on induction. I expect direct proof rather than induction.
- Theorem 8 lacks a rigorous explanation of how the divisibility property is maintained across different indices.

**Computational Verification:**

- The paper states that GNU Octave was used for numerical verification but does not present any computational results. A section summarizing numerical experiments would strengthen the claims.
- The relationship with the golden ratio is interesting, but the claim that "Mulatu numbers can be used to calculate the golden ratio" should be better substantiated with precise mathematical arguments.

**Language and Formatting Issues:**

- Several grammatical errors appear throughout the text, such as:
  - "These findings play a vital role in the boarder context of mathematical sequences" (should be "broader").
  - "We have also shown that, similar to Fibonacci’s numbers, Mulatu’s numbers also give the so-called golden ratio" (redundant use of "also").
- The paper should maintain a more formal tone and avoid phrases like "fascinating patterns" without proper justification.
- The formatting of mathematical expressions is inconsistent, particularly in the placement of summations and recurrence relations.

**References:**

- Several references lack proper formatting. Please organize them in a certain way. 

**Overall Recommendation:** While the paper introduces interesting results related to Mulatu numbers, there are significant issues with the rigour of proofs, mathematical notation, and clarity of exposition. The authors should:

1. Strengthen the proofs with clearer justifications and better-structured induction arguments.
2. Provide numerical verification where computational methods are referenced.
3. Improve the language and formatting to enhance readability.
4. Clarify the motivation behind the new definitions and properties introduced.

After these major revisions, the paper would be more suitable for peer review and indexing.

Is the work clearly and accurately presented and does it cite the current literature?

No

If applicable, is the statistical analysis and its interpretation appropriate?

Not applicable

Are all the source data underlying the results available to ensure full reproducibility?

Yes

Is the study design appropriate and is the work technically sound?

Yes

Are the conclusions drawn adequately supported by the results?

Yes

Are sufficient details of methods and analysis provided to allow replication by others?

No

Reviewer Expertise:

Number Theory

I confirm that I have read this submission and believe that I have an appropriate level of expertise to confirm that it is of an acceptable scientific standard, however I have significant reservations, as outlined above.

# Response

**Published:** 2025-03-03

**Authors:** AGEZE ADMASU

**Affiliations:** Mathematics, Woldia University, Woldia, Amhara, Ethiopia

Dear Professor **Priyabrata Mandal,**

 I hope this message finds you well. I wanted to take a moment to express my sincere gratitude for the time and effort you dedicated to reviewing my article, **More on the fascinating characterizations of Mulatu’s numbers** **. **Your thoughtful and constructive comments have been incredibly valuable in improving the quality of the work.

 I particularly appreciate the insights you provided deeply. Your feedback helped me see new perspectives and refine key sections of the manuscript. The suggestions you offered were both insightful and thorough, and I am confident that the article has greatly benefitted from your expertise.

 Once again, thank you for your careful review and constructive input. Your contributions are highly valued, and I am grateful for the support you’ve given in helping me improve my work.

 With regards,

 Ageze Abye Admasu

 Ageze.ab19@gmail.com

**Point-by-point response**

 Manuscript title: **More on the fascinating characterizations of Mulatu’s numbers**

 DOI: 10.12688/f1000research.157738.1

 First of all, we want to thank the reviewers and editors sincerely for their insightful and encouraging comments.

**Reviewer Comments and our response**

*Title and Abstract*

**Reviewer Comments**

 •The title is appropriate and reflects the paper's content.

 •The abstract is clear but somewhat not standard. The phrase "beautiful and incredible patterns" is informal for an academic paper. A more precise statement would improve clarity.

**Our Response**

 Thank you, and sorry for the inconvenience

 All comments given here are accepted and corrected.

*Introduction:*

**Reviewer Comments**

- The introduction does not adequately define the novelty of the work. The discussion on the relationship between Mulatu numbers, Fibonacci numbers, and the golden ratio should be more structured.

**Our Response**

 Thank you, we modified it.

*Mathematical Rigor and Errors:*

**Reviewer Comments**

- Lemma 1 states, "Any two consecutive Mulatu numbers are relatively prime." A proof or reference is missing.
- In the proof of Theorem 1, the table for j,x, and y is completely unnecessary. The author should identify explicitly what those x and y actually mean. By this, I mean to show that M _i+j_=F _j-1 _M _i_ + F _j_ M _i+1_, where F _n_ denotes the nth Fibonacci number. 
- The proof of Theorem 2 contains an induction step that is not fully justified. There is no need for induction here; just use the recurrence relation for M _n_​​​​​​.
- In Theorem 3, the proof is poorly structured, making it difficult to follow the logic behind the claim. No need for induction; note that M _2n_=(M _2n+1 _- M _2n-1_), and proceed.
- Several theorems, such as Theorems 6 and 7, rely on induction. I expect direct proof rather than induction.
- Theorem 8 lacks a rigorous explanation of how the divisibility property is maintained across different indices.

**Our Response**

 Noted!

 Accordingly, we try to address each comment on the revised manuscript as follows:

- Lemma 1 is proved.
- Theorem 1, 2 and 3 are proved using the comments.
- Apricating your view, the proofs of theorem 6 and 7 using recursion are relatively longer to publish on this manuscript and it will not compromise the quality of the paper.
- Theorem 8 is clarified.

*Computational Verification*

**Reviewer Comments**

- The paper states that GNU Octave was used for numerical verification but does not present any computational results. A section summarizing numerical experiments would strengthen the claims.

**Our Response**

 We appreciate your view. However, the use of GNU Octave was only to conceptualize hypothesis of theorems by taking numerical examples before formal proofs are done. We have also revised the manuscript to state this clearly. 

**Reviewer Comments**

- The relationship with the golden ratio is interesting, but the claim that "Mulatu numbers can be used to calculate the golden ratio" should be better substantiated with precise mathematical arguments.

**Our Response**

 We apologize for the inconvenience. It is edited.

*Language and Formatting Issues*

**Reviewer Comments**

- Several grammatical errors appear throughout the text, such as:
  - "These findings play a vital role in the boarder context of mathematical sequences" (should be "broader").
  - "We have also shown that, similar to Fibonacci’s numbers, Mulatu’s numbers also give the so-called golden ratio" (redundant use of "also").
- The paper should maintain a more formal tone and avoid phrases like "fascinating patterns" without proper justification.
- The formatting of mathematical expressions is inconsistent, particularly in the placement of summations and recurrence relations.

**Our Response**

 Thank you, and sorry for your inconvenience

 All comments given here are accepted and corrected.

*References*

**Reviewer Comments**

- Several references lack proper formatting. Please organize them in a certain way. 

**Our Response**

 Thank you, and sorry for the inconvenience. We have revised references on the manuscript accordingly.

*Overall Recommendation*

**Reviewer Comments**

 While the paper introduces interesting results related to Mulatu numbers, there are significant issues with the rigour of proofs, mathematical notation, and clarity of exposition. The authors should:

1. Strengthen the proofs with clearer justifications and better-structured induction arguments.
2. Provide numerical verification where computational methods are referenced.
3. Improve the language and formatting to enhance readability.
4. Clarify the motivation behind the new definitions and properties introduced.

**Our Response**

 All comments given here are accepted and corrected accordingly.

*Thank you, reviewers!!*

*We got many constructive comments and suggestions which helps to further development of the manuscript and experience for our future work.*
