# Supplementary material for: More on the Fascinating Characterizations of Mulatu’s Numbers
Source: F1000Res. 2025 Apr 17;13:1306. Originally published 2024 Oct 31. [Version 2] doi: 10.12688/f1000research.157738.2 (PMC12963824; doi:10.12688/f1000research.157738.2)
Supplement: Peer review history

# Response

**Published:** 2025-03-03

**Authors:** AGEZE ADMASU

**Affiliations:** Mathematics, Woldia University, Woldia, Amhara, Ethiopia

Dear Professor **Priyabrata Mandal,**

 I hope this message finds you well. I wanted to take a moment to express my sincere gratitude for the time and effort you dedicated to reviewing my article, **More on the fascinating characterizations of Mulatu’s numbers** **. **Your thoughtful and constructive comments have been incredibly valuable in improving the quality of the work.

 I particularly appreciate the insights you provided deeply. Your feedback helped me see new perspectives and refine key sections of the manuscript. The suggestions you offered were both insightful and thorough, and I am confident that the article has greatly benefitted from your expertise.

 Once again, thank you for your careful review and constructive input. Your contributions are highly valued, and I am grateful for the support you’ve given in helping me improve my work.

 With regards,

 Ageze Abye Admasu

 Ageze.ab19@gmail.com

**Point-by-point response**

 Manuscript title: ** More on the fascinating characterizations of Mulatu’s numbers**

 DOI: 10.12688/f1000research.157738.1

 First of all, we want to thank the reviewers and editors sincerely for their insightful and encouraging comments.

**Reviewer Comments and our response**

*Title and Abstract*

**Reviewer Comments**

 •The title is appropriate and reflects the paper's content.

 •The abstract is clear but somewhat not standard. The phrase "beautiful and incredible patterns" is informal for an academic paper. A more precise statement would improve clarity.

**Our Response**

 Thank you, and sorry for the inconvenience

 All comments given here are accepted and corrected.

*Introduction:*

**Reviewer Comments**

- The introduction does not adequately define the novelty of the work. The discussion on the relationship between Mulatu numbers, Fibonacci numbers, and the golden ratio should be more structured.

**Our Response**

 Thank you, we modified it.

*Mathematical Rigor and Errors:*

**Reviewer Comments**

- Lemma 1 states, "Any two consecutive Mulatu numbers are relatively prime." A proof or reference is missing.
- In the proof of Theorem 1, the table for j,x, and y is completely unnecessary. The author should identify explicitly what those x and y actually mean. By this, I mean to show that M _i+j_=F _j-1 _M _i_ + F _j_ M _i+1_, where F _n_ denotes the nth Fibonacci number. 
- The proof of Theorem 2 contains an induction step that is not fully justified. There is no need for induction here; just use the recurrence relation for M _n_​​​​​​.
- In Theorem 3, the proof is poorly structured, making it difficult to follow the logic behind the claim. No need for induction; note that M _2n_=(M _2n+1 _- M _2n-1_), and proceed.
- Several theorems, such as Theorems 6 and 7, rely on induction. I expect direct proof rather than induction.
- Theorem 8 lacks a rigorous explanation of how the divisibility property is maintained across different indices.

**Our Response**

 Noted!

 Accordingly, we try to address each comment on the revised manuscript as follows:

- Lemma 1 is proved.
- Theorem 1, 2 and 3 are proved using the comments.
- Apricating your view, the proofs of theorem 6 and 7 using recursion are relatively longer to publish on this manuscript and it will not compromise the quality of the paper.
- Theorem 8 is clarified.

*Computational Verification*

**Reviewer Comments**

- The paper states that GNU Octave was used for numerical verification but does not present any computational results. A section summarizing numerical experiments would strengthen the claims.

**Our Response**

 We appreciate your view. However, the use of GNU Octave was only to conceptualize hypothesis of theorems by taking numerical examples before formal proofs are done. We have also revised the manuscript to state this clearly. 

**Reviewer Comments**

- The relationship with the golden ratio is interesting, but the claim that "Mulatu numbers can be used to calculate the golden ratio" should be better substantiated with precise mathematical arguments.

**Our Response**

 We apologize for the inconvenience. It is edited.

*Language and Formatting Issues*

**Reviewer Comments**

- Several grammatical errors appear throughout the text, such as:
  - "These findings play a vital role in the boarder context of mathematical sequences" (should be "broader").
  - "We have also shown that, similar to Fibonacci’s numbers, Mulatu’s numbers also give the so-called golden ratio" (redundant use of "also").
- The paper should maintain a more formal tone and avoid phrases like "fascinating patterns" without proper justification.
- The formatting of mathematical expressions is inconsistent, particularly in the placement of summations and recurrence relations.

**Our Response**

 Thank you, and sorry for your inconvenience

 All comments given here are accepted and corrected.

*References*

**Reviewer Comments**

- Several references lack proper formatting. Please organize them in a certain way. 

**Our Response**

 Thank you, and sorry for the inconvenience. We have revised references on the manuscript accordingly.

*Overall Recommendation*

**Reviewer Comments**

 While the paper introduces interesting results related to Mulatu numbers, there are significant issues with the rigour of proofs, mathematical notation, and clarity of exposition. The authors should:

1. Strengthen the proofs with clearer justifications and better-structured induction arguments.
2. Provide numerical verification where computational methods are referenced.
3. Improve the language and formatting to enhance readability.
4. Clarify the motivation behind the new definitions and properties introduced.

**Our Response**

 All comments given here are accepted and corrected accordingly.

*Thank you, reviewers!!*

*We got many constructive comments and suggestions which helps to further development of the manuscript and experience for our future work.*
